# Copper(I) Iodide Thin Films: Deposition Methods and Hole-Transporting Performance

**DOI:** 10.3390/molecules29081723

**Published:** 2024-04-11

**Authors:** Mahboubeh Jamshidi, James M. Gardner

**Affiliations:** Department of Chemistry, Division of Applied Physical Chemistry, KTH Royal Institute of Technology, SE-10044 Stockholm, Sweden

**Keywords:** copper iodide, hole-transport material, deposition methods, solar cells, thin-film transistors

## Abstract

The pursuit of p-type semiconductors has garnered considerable attention in academia and industry. Among the potential candidates, copper iodide (CuI) stands out as a highly promising *p*-type material due to its conductivity, cost-effectiveness, and low environmental impact. CuI can be employed to create thin films with >80% transparency within the visible range (400–750 nm) and utilizing various low-temperature, scalable deposition techniques. This review summarizes the deposition techniques for CuI as a hole-transport material and their performance in perovskite solar cells, thin-film transistors, and light-emitting diodes using diverse processing methods. The preparation methods of making thin films are divided into two categories: wet and neat methods. The advancements in CuI as a hole-transporting material and interface engineering techniques hold promising implications for the continued development of such devices.

## 1. Introduction

Inorganic transparent semiconductors are required in solar cells and other devices, like thin-film transistors (TFTs), light-emitting diodes (LEDs), and smart sensors [1,2,3,4,5,6]. Solar cells produce electric power from solar energy and are a promising option to meet the energy demand with high efficiency. In the last decade, perovskite solar cells (PSCs) have been developed as thin-film solar cells and offer potential advantages over known and long-lasting silicon solar cells, such as being low-cost, lightweight, flexible, semi-transparent, and easy to fabricate through solution processes at low temperatures [7,8]. These advantages have made PSCs a popular subject of research in the field of photovoltaics [9]. Transistors serve as the foundational components in the majority of electronic devices. For decades, ongoing research has focused on producing new varieties of semiconducting materials tailored for thin-film transistors (TFTs). Each time novel transistors or innovative fabrication techniques emerge, numerous applications that were previously inaccessible become achievable. This includes certain advancements, like ultrahigh-definition transparent displays and flexible electronic devices [10,11,12]. At present, light-emitting diodes (LEDs), relying on solid-state semiconductors, have emerged as the next evolution in lighting technology following incandescent and fluorescent lamps. This transition is primarily due to their remarkable stability, exceptional energy efficiency, adjustable color, and ecofriendly attributes, marking a significant milestone in the history of human lighting [13,14].

Hence, there is a considerable need to find materials that possess low resistivity for electrical conduction and high transparency for solar cells, TFTs, and LEDs. This has led to extensive research on compounds, such as In_2_O_3_, ZnO, SnO_2_, and complex oxides [4,15,16,17,18,19]. Despite extensive research, most of these materials discovered thus far display *n*-type conductivity, and there are few viable choices for *p*-type materials with high hole carrier density and/or mobility. This poses a crucial problem, since the practical implementation of electronic circuits relies on the development of both *n*- and *p*-type conducting materials for *p*-*n* junction and complementary circuits, which are essential components [20]. In the quest for conducting transparent semiconducting thin films, only a handful of *p*-type wide band gap materials, such as CuI, CuSCN, and CuAlO_2_, have been discovered [21,22,23,24]. Of these, CuI and CuSCN show great potential for use in dye-sensitized solar cells (DSSC) and extremely thin absorber solar cells [25,26,27,28].

CuI as a non-toxic and water-insoluble material that exhibits three primary polymorphs, namely the cubic α (rock salt structure, Fm3m), hexagonal β (wurtzite structure, P63mc), and cubic γ-phase (zinc blend structure, space group F43m), which changes from the melting point to room temperature (RT) [29,30]. In this temperature range, the α-phase acts as a mixed conductor, with Cu^2+^ ions serving as the primary carrier. The β-phase functions as an ionic conductor, while the γ-phase (below 350 °C) operates as a *p*-type semiconductor, as its conductivity is dependent on the excess stoichiometric iodine presence [31,32,33]. With a broad direct bandgap of 3.1 eV, the energy levels of the valence band maximum (VBM) and conductive band minimum (CBM) are 5.4 and 2.3 eV with respect to the vacuum, respectively, and high exciton binding energy (62 meV). The transparency and hole conductivity of CuI determine its usefulness in various applications, including the construction of fully solid-state dye-sensitized photovoltaic cells, and as a buffer layer in CuInX_2_ (X = S, Se, and Te)- and MoO_3_-based solar cells [34,35,36]. Additionally, it finds application in novel uses, like smart windows (or screens), that incorporate certain functions, such as energy harvesting, cooling, and thermal sensing [37,38,39].

**Figure 1:**
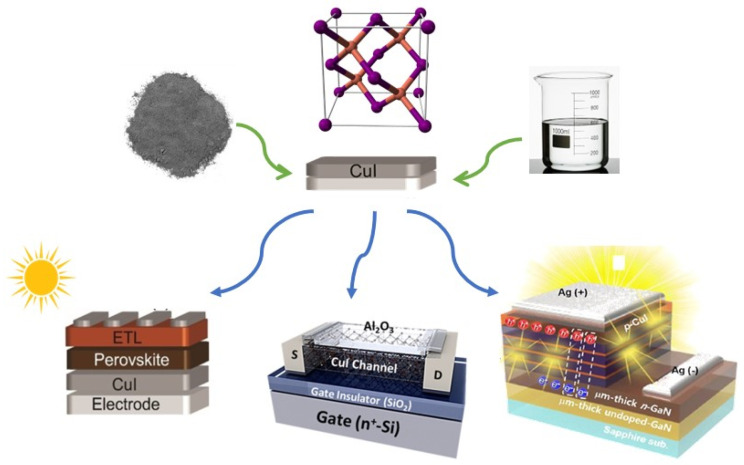
Copper iodide thin films can be produced through both neat and wet methods, from powder CuI and solution of CuI respectively (green arrows), and exhibit functionality in perovskite solar cells (PSCs), thin-film transistors (TFTs), and light-emitting diodes (LEDs) (blue arrows) [40,41].

This review provides a comprehensive summary of the deposition techniques for CuI as a hole-transport material. Based on the presence or absence of solvents during the preparation of the HTL film, this research can be broadly categorized into two groups: solution-processed and neat (dry) thin-film fabrication. Subsequently, it delves into the performance of CuI as a hole-transport layer (HTL) in perovskite solar cells (PSCs), TFTs, and LEDs using various processing methods (Figure 1).

## 2. Deposition Methods of Copper Iodide Thin Film

### 2.1. Solution-Processed Methods

The solution processing nature of wet methods enables them to present distinctive advantages, including low cost, straightforward equipment requirements, and ease of operation [42,43,44]. In the following sections, we will discuss the recent developments in the performance of Cu-based hole-transporting layers (HTLs) using specific wet methods. The performances of some solar cells are demonstrated in Table 1.

#### 2.1.1. Spin Coating

**Figure 2:**
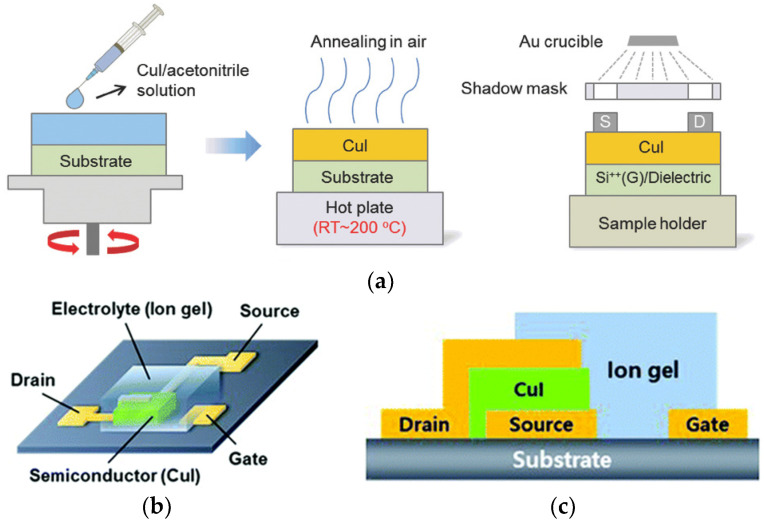
(**a**) Spin-coating process to fabricate CuI TFT [54]; (**b**,**c**) schematic and cross-section of a vertical TFT (VTFT) with a CuI channel layer and an ion gel electrolyte–gate insulator in a side-gated configuration [55].

Spin coating is a widely used technique in the field of optoelectronics for creating thin films in the laboratory [45,46,47]. By applying a precursor solution with a specific concentration onto a rapidly rotating substrate, a uniform film can be formed through the combined influence of centrifugal force and the liquid’s surface tension. The precise control of various processing parameters, such as the choice of solvent, concentration, annealing temperature, and rotation speed, greatly influence the morphology and thickness of films [48,49,50,51,52,53]. A. Liu et al. demonstrated spin-coated inorganic *p*-type copper iodide thin-film transistors (TFTs) with electrical performances strongly related to channel thickness and annealing conditions (Figure 2a) [54]. Acetonitrile was a crucial solvent due to its highly volatility, and no residue was left behind. The thickness and annealing conditions plus the heavily doped Si substrate resulted in controlled CuI and gate dielectric thicknesses, respectively. They produced room-temperature solution-processed *p*-type CuI/ZrO_2_ TFTs with impressively high µ_FE_ (1.93 cm^2^ V^−1^ s^−1^) at an operating voltage of 5 V.

Han Ju Lee et al. reported CuI films on glass and polyethylene terephthalate (PET) substrates with a 120 nm thickness [20]. CuI powder was dissolved in dipropyl sulfide and stirred for 6 h at 60 °C, and then spin-coated onto the PET substrate (1200 rpm for 60 s). The resulting films were subsequently annealed in ambient air for 1 h. The thin film displayed a notable hole carrier density of 9.8 × 10^18^ cm^−3^ and a sheet resistance measuring of 2.2 kΩ sq^−1^, indicating its *p*-type conductivity. Subsequently, CuI films were utilized as both a source-drain electrode in thin-film transistors and a sensing material in strain sensors.

It is noteworthy that although most applications are limited to γ-CuI, H. J. Lee et al. reported using amorphous CuI semiconductors as the channel layer, with electrolyte-gated p-channel TFTs in a vertical device structure (Figure 2b,c). [55] The precursor solution was prepared by dissolving CuI powder in two co-solvents, namely acetonitrile–ethanolamine (77/23 vol%) and methoxyethanol–ethanolamine (57/43 vol%). The fabricated TFTs exhibited excellent electrical performance, with high current densities above 1000 mA cm^−2^ at VG = −0.4 V, ON/OFF current ratios of up to 3 × 10^4^, and high transconductances of up to 6.46 S m^−1^.

**Figure 3:**
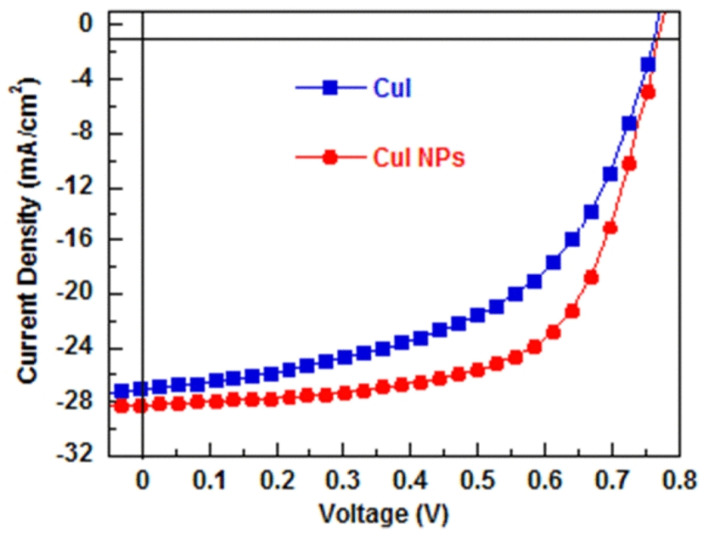
*J*–*V* characteristics of PSCs with CuI and CuI NPs as HTLs under the illumination of AM1.5G, 100 mW/cm^2^ [56].

Yuancong Zhong et al. applied CuI as HTL in polymer solar cells with the configuration of ITO/CuI/P3HT:PC_61_BM/Ca/Al. Their synthesis of CuI nanoparticles (NPs) involved the decomposition of a CuI *N,N*−dimethylformamide (DMF) dispersion using water [56]. Subsequently, a solution containing CuI NP dispersed in acetonitrile was spin-coated onto the surface of ITO, serving as the hole-transport layer (HTL). As a result, the CuI NP film exhibited uniform surface coverage and minimal surface roughness. The use of CuI NPs as the HTL in polymer solar cells led to improved power conversion efficiency (PCE) and stability compared to using bulk CuI as the HTL (Figure 3).

**Figure 4:**
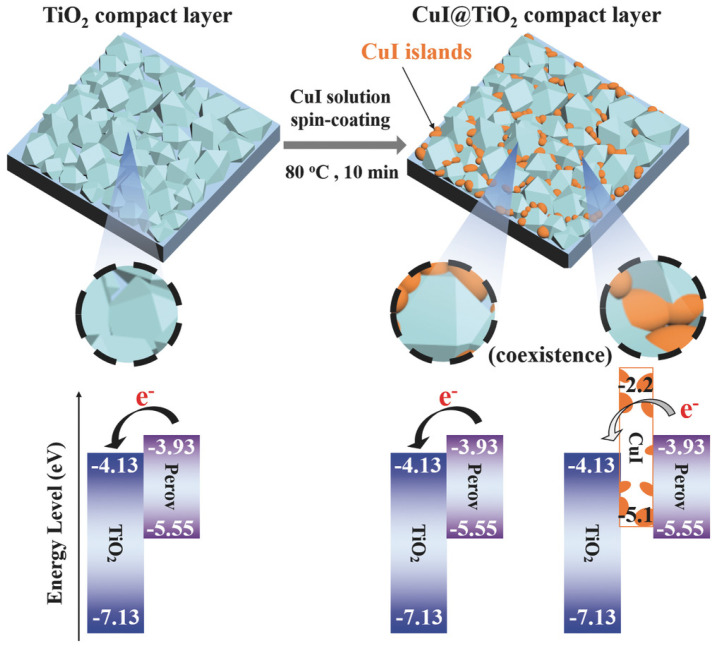
A diagram illustrates the process of CuI island formation on the TiO_2_ compact layer, followed by the deposition of a perovskite layer. In devices based on CuI-modified TiO_2_ (CuI@TiO_2_), electrons originating from the perovskite layer migrate towards the exposed surface of TiO_2_ but are unable to transfer to the CuI islands due to the significantly higher energy level of its conduction band [57].

M. M. Byranvand et al. used CuI in planar perovskite solar cells where TiO_2_ is usually used as an electron transport material [57]. However, the unsuitable conduction band energy and low electron extraction ability of TiO_2_ resulted in low efficiency cells. They modified the electron transport layer with CuI via a simple spin-coating process, leading to a PCE of 19.0% with high reproducibility and negligible *J–V* hysteresis. The enhancement occurs due to the inherent *p*-type characteristics of CuI, which attract electrons towards the interface of perovskite and TiO_2_. This leads to a repositioning of energy levels and enables efficient extraction of electrons while minimizing the presence of traps (Figure 4).

In another article CuI-doped 2,2,7,7-tetrakis(*N*,*N*−di-*p*-methoxyphenylamine)-9,9-spirobifluorene (spiro-OMeTAD) was used as the HTL, and PCE was improved from 14.82% to 18.02% [58]. This was due to obvious enhancements in the cell parameters of short-circuit current density and fill factor. In addition to its role as a hole-transport material (HTM), the composite film also acts as a barrier, effectively preventing film aggregation and crystallization of spiro-OMeTAD films. This results in a reduction in pinholes and voids, which helps to slow down the decomposition of perovskite by limiting the infiltration of moisture to a certain extent.

In 2022, A. Choudhur et al. used nanocrystalline copper iodide (CuI) as a hole-injection and -transport material for the fabrication of organic LEDs [59]. They compared the performance of the new LED based on nanocrystalline CuI with an amorphous and PEDOT:PSS-based device. Superior performance was obtained, with an EQE_max_ of 17%, a PE_max_ of 64 lm W^−1^, and a CE_max_ of 62 cd A^−1^.

#### 2.1.2. Spray Coating

**Figure 5:**
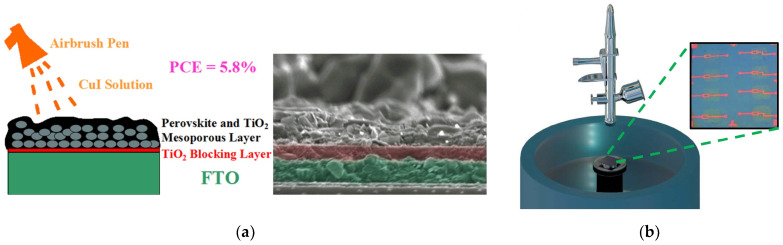
(**a**) Schematic of airbrushing the CuI solution and SEM image of interfaces among each layer in the FTO/TiO_2_/CH_3_NH_3_PbI_3−x_Cl_x_/CuI/Au solar cell [60]; (**b**) schematic diagram of the spray–spin coating method and optical image of the active layer patterned TFTs [62].

The spray deposition technique has certain advantages over both gas-phase and liquid-phase methods, making it a versatile technology for film production. The spray coating method uses an airbrush to spray a solution on the substrate. The thickness of the films is controlled by the amount of solution sprayed onto the substrate. Moreover, the method provides the possibility to fabricate large area cells. M. Huangfu et al. reported the first CuI thick film prepared by spray process as HTL in planar heterojunction-based FTO/TiO_2_/CH_3_NH_3_PbI_3−x_Cl_x_/CuI/Au solar cells with a PCE of 5.8% (Figure 5a) [60].

X. Li et al. reported a film which could reach a maximum photovoltaic efficiency of 17.6% with reduced hysteresis in perovskite solar cells (PVKs) where 60 nm thick CuI layer HTM was spray-coated based on the Na-modified TiO_2_ electron transport layer (ETL) [61].

#### 2.1.3. Spray–Spin Coating

The spray–spin coating method is a combination of spray and spin coatings. The prepared solution is sprayed while the substrate is rotating inside a spin coater (Figure 5b). K. Lee et al. patterned Zn-doped CuI (Zn:CuI) using this method to fabricate *p*-type TFTs where only γ-phase CuI crystals were grown [62].

#### 2.1.4. Drop Casting

Drop casting, as a straightforward technique for film fabrication, aligns more closely with coating processes relevant to industry. The self-spreading behavior observed during drop casting arises from the uneven surface tension between the solution/substrate, solution/air, and air/substrate interfaces [63]. This distinctive characteristic enables the formation of films without requiring specialized deposition equipment. Moreover, the drop casting method not only saves time and materials but also shares similarities with industry-relevant coating techniques, such as slot-die coating, in terms of fluid dynamics and drying kinetics. This makes it a promising alternative for producing films from a specific precursor formulation [64,65].

**Figure 6:**
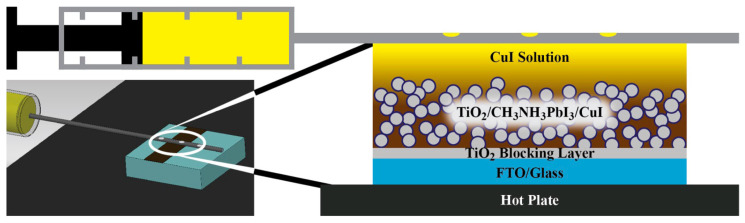
Automated drop casting apparatus for the solution deposition of CuI onto mesoporous TiO_2_/CH_3_NH_3_PbI_3_ films [66].

Christians et al. reported the deposition of CuI solution by the drop casting method [66]. CuI was dissolved in a mixture of *n*-propyl sulfide−chlorobenzene solvents. As is shown in Figure 6, a syringe with a perforated needle in the upper section was positioned above the 80 °C TiO_2_/CH_3_NH_3_PbI_3_ film. An automated mechanical motor was employed to move a droplet of CuI solution across the substrate. This facilitated the uniform deposition of CuI solution over the entire solar cell, effectively filling the porous TiO_2_/CH_3_NH_3_PbI_3_ structure and creating a CuI layer with a thickness of around 1.5−2.0 μm. Consequently, the power conversion efficiency (PCE) of the CuI-based perovskite solar cells (PSCs) reached 6.0%.

#### 2.1.5. Successive Ionic Layer Adsorption and Reaction (SILAR) Technique

**Figure 7:**
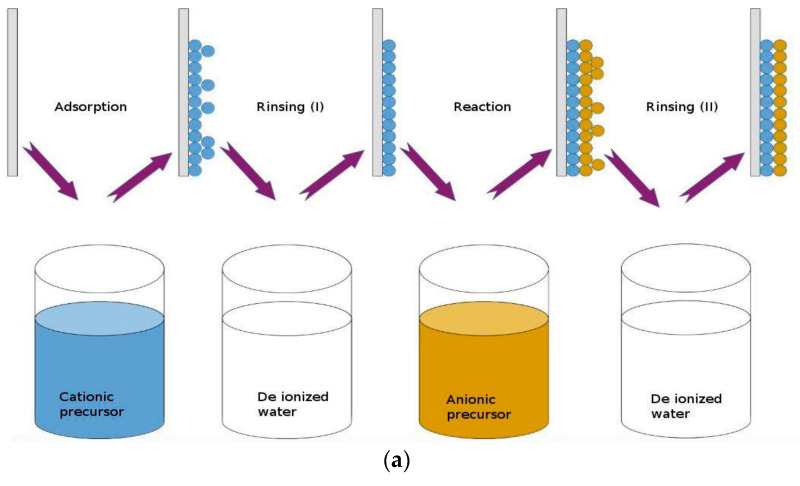

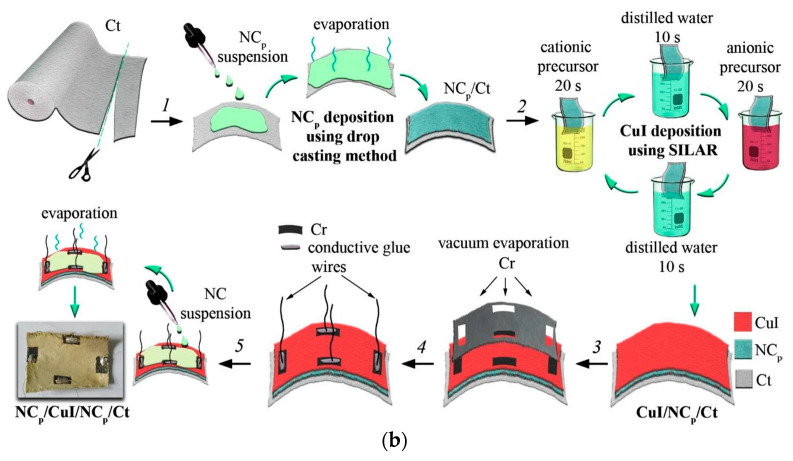
(**a**) Schematic depiction of the thin film formation using the SILAR method [68]; (**b**) schematic representation of the fabrication steps for the thermoelectric module utilizing TE textile NCp/CuI/NCp/Ct, featuring Cr contacts for external circuit connection via conductive glue, alongside a photograph showcasing the completed thermoelectric module [69].

SILAR was established by Y.F. Nicolau at 1985 with the intention of growing polycrystalline or epitaxial thin films of water-insoluble ionic or ion-covalent compounds [67]. SILAR is a straightforward, inexpensive, and scalable method for large scale depositions on a variety of substrates. The method involves immersing the substrate in a solution containing a soluble salt of the desired cation where cations get adsorbed by attractive physical forces on the substrate. It is followed by immersion in a solution containing a soluble salt of the desired anion of the compound to be reacted with cations and grown. After each immersion, the substrate carrying the developing film is thoroughly rinsed in high-purity deionized water to remove the un-adsorbed ions and loosely bonded ions, respectively (Figure 7a). Such cycles are repeated till the film reaches a considerable thickness.

SILAR has been utilized for room temperature (25 °C) and ambient pressure deposition of *p*-type CuI thin films from an aqueous environment onto glass and copper substrates to produce a zinc blend structure with a γ-phase [70]. The cationic precursor contains a copper CuSO_4_ (0.1 mol/dm^3^) solution mixed with sodium thiosulphate, Na_2_S_2_O_3_, (0.1 mol/dm^3^) as a ligand and reducing agent to convert Cu(II) to Cu(I). For the formation of CuI films, iodide in the form of potassium iodide serves as the respective anion source [25].

The molar ratio of CuSO_4_:Na_2_S_2_O_3_, repetition and length of time for each cycle, and the substrate influence the homogeneity and thickness of the film. For instance, a more uniform surface coverage and a lower surface roughness are observed for CuI film on FTO compared to on glass. By controlling the soaking time (5 s Cu^+2^:5 s water:20 s I^−^:5 s water) and performing 25 cycles, CuI film with a thickness of 250–300 nm and a 75% transmission in the visible region was obtained [25]. In another report, only the time of rinsing the substrate in double distilled water (3 s) and the number of SILAR cycles (30) changed, which resulted in a thicker film that tended to peel off from the substrate after 80 cycles [70].

In 2020, N.P. Klochko et al. used the SILAR method to deposit nanostructured CuI thin films on flexible, biodegradable nanocellulose (NC) substrates to serve as protection against terrestrial ultraviolet (UV) radiation from the solar spectrum [71]. The results indicated that the UV-protection capacity of CuI films on NC substrates falls within the “excellent” category, with sun protection factors reaching up to 9211 for the finest CuI film produced via the SILAR method. Additionally, nanostructured ZnO and CuI thin films on poly(ethylene terephthalate) tapes demonstrated effective UV-shielding applications [72].

In 2021, another study by N.P. Klochko et al. added the polymer nanocellulose (NC_p_) sublayer on the CuI film with an average thickness of 10 µm, within the wearable thermoelectric textile. The developed module demonstrated an impressive output power density of 44 µW/cm^2^ at a temperature gradient of 50 K, which is one of the best results currently known for solid, miniature, flexible, and fabric- and textile-based thermoelectric generators (TEGs) [69]. The process of fabrication and SILAR method of CuI is illustrated in Figure 7b.

#### 2.1.6. Chemical Bath Deposition (CBD) Technique

**Figure 8:**
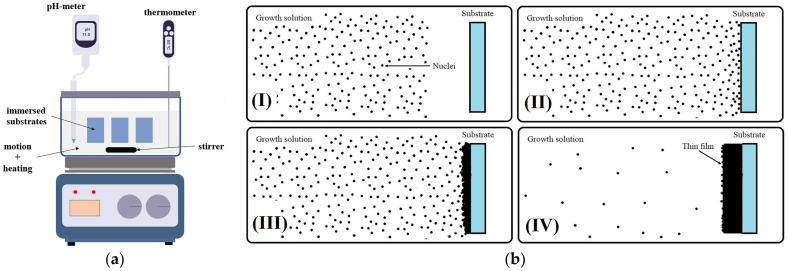
(**a**) Experimental arrangement for the chemical bath deposition (CBD) method; (**b**) diagram illustrating the progression of nanomaterial growth via chemical bath deposition (CBD) over time. (**I**) Initiation of nuclei formation, (**II**) migration of nuclei towards the substrate surface, (**III**) aggregation of nuclei and initiation of thin film growth, and (**IV**) saturation of the deposition solution marking the completion of growth [74].

The technique of chemical bath deposition (CBD) is a popular method utilized for the fabrication of thin films, encompassing a range of thicknesses from a few nanometers to multiple microns. Despite its initial introduction being relatively obscure, the technique can be traced back to 1869 [73]. This method of growth stands out as a straightforward, cost-effective, and highly manageable approach. It can be conceptualized as a process wherein the product is both generated and deposited within a single location, namely the chemical bath, which can be set up with ease (Figure 8a). The CBD method utilizes an aqueous solution under mild conditions (usually below 100 °C) and normal atmospheric pressure, with distilled or deionized water serving as the host solvent. Ensuring control over essential parameters during the chemical bath process is relatively simple and adheres to a systematic methodology.

As illustrated in Figure 8b, the initial step primarily involves the generation of species, driven predominantly by the ion-by-ion or cluster-by-cluster mechanism, leading to an increase in material quantity. These processes take place at nucleation sites, which are typically influenced by factors, such as substrate type, quality, and conditions. The generated species then diffuse through a medium and condense onto the substrate. The material is deposited onto substrates submerged in the chemical bath. The thickness of the resulting film is correlated with various parameters, including pH, deposition rate, and bath temperature [74,75].

Regarding CuI film, the same precursors as the SILAR method are used in CBD [76]. An aqueous solution bath is used at room temperature, consisting of CuSO_4_ with pH = 5 and Na_2_S_2_O_3_ as the reducing and complexing agents. With constant stirring, KI with a pH = 6 is added to the flask. After deposition, the samples are taken out, rinsed with water, and then dried with argon gas. The film is formed on the substrate, and precipitate is formed in the chemical bath [25].

#### 2.1.7. Printing Techniques

**Figure 9:**
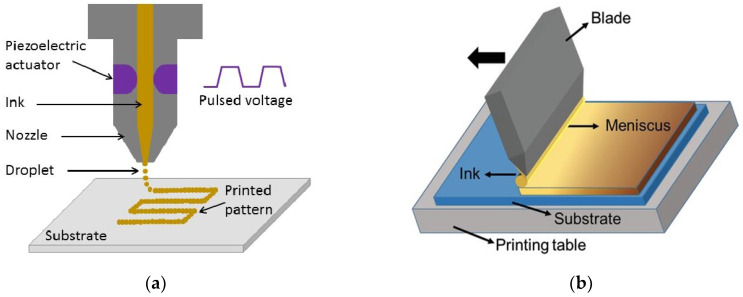

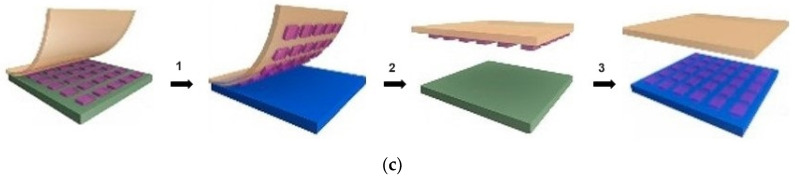
(**a**) Diagram depicting standard inkjet printing systems. An electronically-controlled piezoelectric actuator produces a pressure pulse, ejecting a fluid droplet from the nozzle. Coordination between the electronics and the motion system facilitates the digital creation of intricate designs on flat surfaces [82]; (**b**) schematic illustration of the blade coating technique [83]; (**c**) the transfer printing process typically consists of three sequential steps. Firstly, the materials cultivated on the donor substrate are transferred onto the transfer mediator through the application of pressure, illumination, temperature, or similar methods. Secondly, the transfer mediator, now carrying the transferred materials, is securely bonded to the surface of the acceptor substrate with meticulous control. Lastly, the transfer mediator is removed, leaving the materials behind on the acceptor substrate surface [84].

Printing techniques as solution-processed methods offer advantages, such as scalability, cost-effectiveness, and the ability to deposit materials on various substrates (Figure 9). They play a crucial role in the development and manufacturing of solar cells and displays. However for deposition of CuI, not all of them have been used so far (such as screen printing [77]).

- Inkjet printing

Inkjet printing is a contactless method used for microscale processes and direct deposition of particles (10 nm to 1.0 micron in diameter) onto flexible or rigid substrates. As seen in Figure 9a, this technique enables the creation of patterned films without the need for any masks [78]. Inkjet printing stands out as the most promising method for full-color display applications and due to its capability for large-scale production at low cost [79]. They have also been investigated in PSCs [80]. Chang-Ho Choi et al. reported, for the first time, low-temperature (150 °C) fabrication of printed *p*-type CuI TFTs. Ink containing γ-CuI powder dissolved in acetonitrile solvent was prepared and printed onto the substrate of the device. As the solvent evaporated, the CuI film formed instantly. The printed CuI TFTs at 60 °C exhibited an average field-effect mobility of 1.86 ± 1.6 cm^2^ V^−1^ s^−1^, with the highest value reaching 4.4 cm^2^ V^−1^ s^−1^. They also displayed an average on/off ratio of 10–100, which is comparable to most *p*-type metal oxide TFTs fabricated using solution-based processes that have been reported [81].

- Transfer printing

Transfer printing encompasses a range of methods that enable precise assembly of micro- and nanomaterials, arranging them in spatially organized and functional patterns. These techniques offer versatile pathways, serving not only to fabricate structures and platforms for scientific investigations but also to create high-performance, heterogeneous, and integrated functional systems. These systems find applications in various fields, such as flexible electronics, optoelectronics with three-dimensional and/or curvilinear designs, as well as in bio-integrated sensing and therapeutic devices [85].

The procedure employs a gentle stamp to transfer a layer with a desired thickness (ranging from 10 to 100 s of nm) onto a substrate, and if necessary, to pattern the layer during or after the transfer (Figure 9c). The selection of an appropriate transfer mediator is a crucial aspect of the transfer printing technique to achieve a high yield, exceptional fidelity, and ease of control. In the transfer printing process, transfer mediators, such as organic polymer films, like polydimethylsiloxane (PDMS) and polymethyl methacrylate (PMMA), as well as metal foils, like copper (Cu) and chromium (Cr), have been utilized. However, achieving successful film (its macroscale continuity after printing = the fraction of the film successfully transfer-printed), necessitates careful control of the adhesion/rigidity between the layer and the substrate, as well as between the layer and the stamp [86,87].

Due to restrictions in using CuI dissolved in acetonitrile in *n*–*i*–*p* structured PVK solar cells, which is quite destructive to the perovskite layer, a mixture of di-*n*-propylsulfide/chlorobenzene mixture is used. However, the fast reaction of this mixture with perovskite can affect the perovskite layer. The limitations mentioned above pose constraints on the utilization of CuI HTL in *n*-*i*-*p* structured PSCs. Consequently, the primary obstacle in employing inorganic CuI HTL in PSCs lies in finding a deposition method that does not adversely impact the perovskite layer. In this regard, Ravi P. Srivastava et al. reported the production of perovskite solar cells incorporating CuI as the hole-transport layer (HTL) using a transfer-printing technique, as opposed to employing a (doped) spiro-OMeTAD HTL and costly materials in an *n*–*i*–*p* structure [88]. Perovskite devices processed under standard atmospheric conditions and employing transfer-printed CuI showed a promising efficiency of 8.3%. Although the performance of transfer-printed CuI devices falls short of that achieved by doped spiro-OMeTAD-based PSCs, the produced devices still demonstrate efficiency comparable to undoped spiro-OMeTAD. This suggests a feasible alternative of substituting expensive undoped spiro-OMeTAD with transfer-printed CuI as the HTL.

- Spray printing technique

Using the solution-processable spray printing technique, a remarkably conductive copper iodide (CuI) film exhibiting outstanding thermoelectric (TE) performance was created. This innovative approach enables the fabrication of transparent and flexible thermoelectric generators (TEGs) reported by E. J. Bae et al. [89]. A small amount of I_2_ was incorporated into the CuI precursor solution to avoid sublimation or evaporation-induced iodine loss during the printing process. Two distinct types of flexible thermoelectric generators (TEGs), one in a coiled configuration and the other foldable, were effectively produced. The coiled TEG demonstrated an impressive output power density of 236.5 μW cm^−2^ at a temperature gradient of 22 °C. On the other hand, the foldable TEG exhibited remarkable sensitivity to even minor temperature variations induced by a halogen lamp.

**Table 1:** Photovoltaic performance of solar cells based on the copper iodide as HTL prepared by the solution-processed methods.

Deposition Method	HTM Thickness [nm]	Device Configuration	V_oc_ [V]	J_sc_ [mA cm^−2^]	FF [%]	PCE [%]	Refs.
Spin coating	30	ITO/CuI/CH_3_NH_3_PbI_3_/C60:BCP/Ag	0.99	22.6	71.3	16.8	[90]
	30	ITO/CuI/P3HT:PC_61_ BM/Ca/Al	0.55	9.02	63	3.1	[56]
	30	ITO/CuI NP/P3HT:PC_61_ BM/Ca/Al	0.56	9.71	63.9	3.47	[56]
		ITO/ZnO NP/MEH-PPV:PC_61_ BM/CuI/Ag	1.54	2.5	35	1.35	[91]
Spin coating [NH_3_(aq.)]	30	ITO/CuI/HaP/PCBM/AZO/Ag	0.99	19.37	74	14.21	[92]
Spray coating		FTO/TiO_2_/CH_3_NH_3_PbI_3−x_Cl_x_/CuI/A	0.65	21.0	33	4.5	[60]
	60	FTO/c-TiO_2_/MAPbI_3_/CuI/Au	1.03	22.78	75	17.6	[61]
Drop casting	1500–2000	FTO/TiO_2_/CH_3_NH_3_PbI_3_/CuI/Au	0.55	17.8	62	6.0	[66]
Doctor blading	400	FTO/TiO_2_/CH_3_NH_3_PbI_3_/CuI/graphite/Cu	0.78	16.7	57	7.5	[93]
Transfer printing		ITO/SnO_2_/PVK/CuI/Au	0.79	19.3	0.54	8.3	[88]

- Blade coating (doctor blading)

Blade coating, also referred to as knife coating or bar coating, is a cost-effective and efficient printing technique utilized for large-scale coating applications. It has gained significant popularity as a preferred method for applying thin-film coatings due to its compatibility with both rigid and flexible substrates. In blade coating, a thin film is deposited by directly loading ink onto the substrate and spreading it using a knife-type blade coater. To achieve a uniform wet thin film, either the blade or the substrate is moved, resulting in a controlled thickness (Figure 9b). The meniscus formed by the solution between the blade and the substrate, along with the ink concentration, primarily determines the thickness of the deposited thin film. Parameters, such as the gap between the blade and the substrate, the relative speed of the blade and substrate, ink viscosity, blade geometry, and substrate wettability play crucial roles in controlling the meniscus [83].

G. A. Sepalage et al. employed the doctor blading technique to achieve uniform CuI layers on perovskite layers, resulting in a significant reduction in *J*–*V* hysteresis behavior and a power conversion efficiency (PCE) of 7.5% [93]. The reduced *J–V* hysteresis can be attributed to the rapid polarization relaxation caused by the minimal charge separation at the interface between the perovskite and CuI when coating by this technique. In this method, the damaging effect of polar solvents on the perovskite absorption layer becomes obvious due to the relatively slow evaporation speed of the corrosive solvent.

### 2.2. Neat Methods (Dry Methods)

In addition to solution-processed methods, the uniform CuI film can also be fabricated using neat methods without solvents. In this section, we will present the latest developments in interface engineering of CuI hole-transport layers using dry methods. These methods primarily include powder pressing and physical vapor deposition techniques.

#### 2.2.1. Powder Pressing

**Figure 10:**
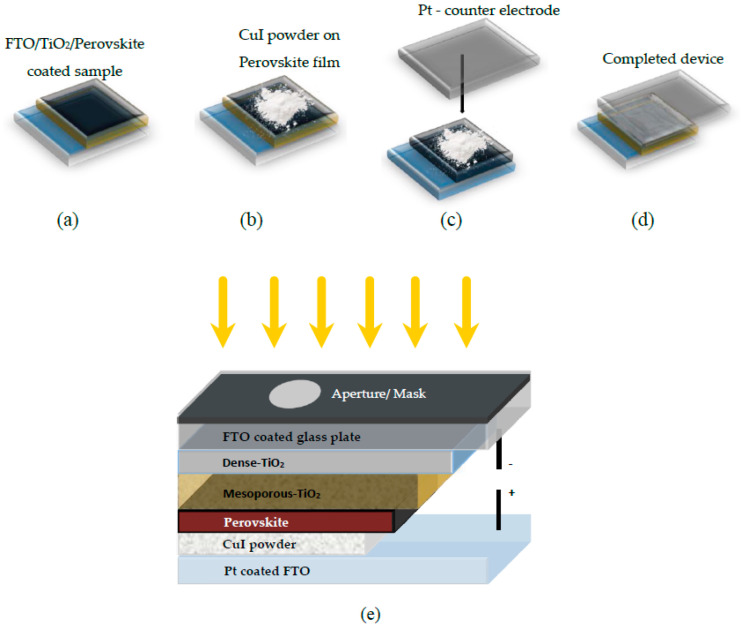
(**a**–**d**) Sequential diagram illustrating the powder pressing technique for integrating CuI powder as the hole-transport material (HTM) in perovskite solar cells (PSCs), and (**e**) schematic depiction of the CuI device architecture with yellow arrows as sunshine [94].

A simple powder press vacuum-free technique for the deposition of CuI, which involves a direct introduction of CuI powder over the perovskite film, was applied by S. Uthayaraj et al. [94]. CuI powder was layered between the perovskite layer and Pt top-contact (Figure 10). When the device was subjected to an illumination of 100 mW/cm^2^ with an air mass (AM) 1.5 filter in the air, the CuI devices exhibited an average short-circuit current density (J_SC_) of over 24 mA/cm^2^, slightly surpassing that of the spiro-OMeTAD device. The improved performance of the devices can be attributed to the higher hole mobility of CuI, which reduces electron–hole recombination. However, devices utilizing CuI as the hole-transport material (HTM) demonstrated limited performance, characterized by lower open-circuit voltage (0.66 ± 0.02 V) and fill factor (FF) (0.49 ± 0.03) compared to those utilizing spiro-OMeTAD as HTMs (VOC = 0.79 ± 0.03 V and FF of 0.56 ± 0.07). The reduced fill factor in CuI devices is likely associated with the increased thickness and roughness of the pressed CuI layer. However, achieving precise control over the thickness of CuI using the pressing method requires careful optimization of the process parameters and addressing challenges related to uniformity and scalability.

X. Han et al. synthesized CuI powder and fabricated the CuI film using vacuum-assisted filtration of the CuI powder onto a porous nylon membrane, followed by a hot pressing step for application in thermoelectric (TE) materials used to convert heat into electrical energy [95]. Furthermore, the film exhibits impressive flexibility, with approximately 95% of its electrical conductivity retained after undergoing 1000 bends along a rod with a 4 mm radius. During a finger touch test conducted on a single-leg TE module, it was noted that a voltage of 0.9 mV was quickly generated within 0.5 s in reaction to a 4 K temperature variance between a finger and the ambient surroundings. This observation suggests encouraging prospects for application in wearable thermal sensors.

#### 2.2.2. Physical Vapor Deposition Techniques (PVD)

**Figure 11:**
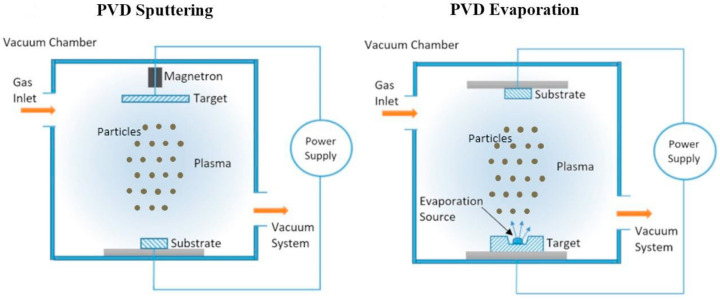
Illustration of the PVD; magnetron sputtering and evaporation coating methods [96].

Physical vapor deposition (PVD) is a process of vacuum deposition where the material transitions from a solid phase to a vapor phase and subsequently reverts to a solid phase, forming a thin film. Sputtering and evaporation stand out as the primary PVD techniques employed in photovoltaic (PV) manufacturing (Figure 11). For deposition of CuI by PVD techniques, there are two major methods: (1) deposit CuI directly onto the substrate and (2) first deposit Cu on the substrate and then use a solid–gas reaction to convert it to CuI.

- Thermal evaporation

The thermal evaporation method is considered a gentler process, causing less damage to the substrate and low pollution. It enables a very high degree of control over film thickness and morphology, so it produces continuous and reproducible films. However, this technique is expensive and difficult to scale up since its scalability to large-scale production is limited by cost considerations, energy consumption, material wastage, complexity of operation, and maintenance requirements. In the procedure, a measured amount of CuI powder is put in the evaporation boat in the vacuum thermal evaporator machine at room temperature. K. P. Marshall et al. evaporated CuI as HTL in a *p*–*i*–*n* perovskite photovoltaics (PPV) device by thermal evaporation based on a CuI/CsSnI_3_/fullerene planar layer architecture [97].

**Figure 12:**
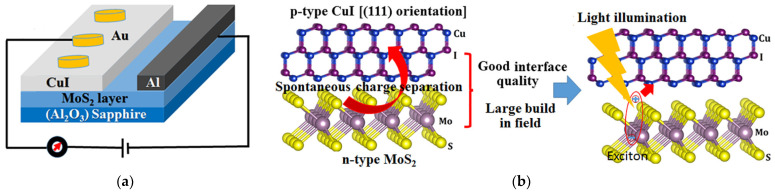
(**a**) Diagram illustrating the γ-CuI/MoS_2_ heterojunction device. (**b**) Crystal structures viewed in cross-section of cubic γ-CuI along the (111) plane atop MoS_2_ layers; the presence of γ-CuI (111) facilitates the establishment of a productive heterojunction interface, conducive to exciton dissociation and separation [98].

R. D. Mahyavanshi et al. made a photoresponsive heterojunction device by deposition of CuI on the molybdenum disulfide (MoS_2_) layer by vacuum thermal evaporator (Figure 12). Photoluminescence quenching and an excellent photoresponsivity of 0.27 A W^−1^ at a bias voltage of 5 V with the illumination of monochromatic light were obtained [98].

**Figure 13:**
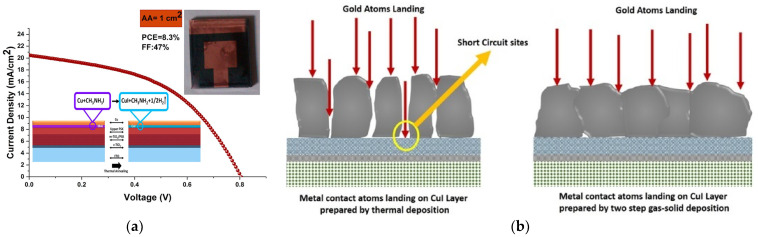
(**a**) Illustration depicting the CuI formation interaction at the interface between the CH_3_NH_3_PbI_3_ and Cu layers [99]; (**b**) depiction of the migration of Au atoms through the pinholes in the thermally deposited CuI and CuI produced via the gas–solid method [100].

P. Nazari et al. used interface engineering to grow CuI in a solar cell. They performed thermal deposition of Cu in a PVK solar cell in which copper reacts at the interface of the perovskite and Cu layer with the excess iodine in methylammonium iodide (MAI) and Cu atoms. The fabricated solar cells show 9.24% and 8.3% efficiencies for 0.1 and 1 cm^2^ active areas, respectively (Figure 13a) [99]. In another report, S. Gharibzadeh et al. evaporated Cu on the perovskite, and using gas–solid phase transformation they converted Cu to CuI [100]. They compared the thermally deposited CuI and CuI obtained using the gas–solid method. Typically, during the one-step deposition of CuI through thermal deposition or sputtering, columnar microstructures are formed, and these structures may contain pinholes. These pinholes have the potential to serve as diffusion sites for atoms from the upper Au contact layer. However, in the latter technique, there is a remarkable improvement in uniformity and coverage. This leads to a reduction in pinholes and minimizes the direct contact between the electrodes and the TiO_2_ sublayer (Figure 13b). Moreover, an extraordinarily high short-circuit current density of 32.72 mA cm^−2^ is achieved, which is attributed to the superior quality of the deposited CuI layer.

K. Ahn reported synthesizing *p*-type sulfur-doped CuI (CuI:S) thin film using a liquid-iodination method with a thiol additive [101]. It was effectively utilized as transparent conductive electrodes in green organic LEDs and as a *p*-type transparent thin-film transistor.

M. Wang et al. demonstrated a simple and environmentally friendly method to grow large-area *p*-type conductive CuI films (resistivity of 0.02 Ω cm, transmittance of 73%) by thermally evaporating copper and then dipping it into an ethanol solution of iodine at room temperature. They showed that the iodine concentration is the most important factor for the growth of CuI films [102].

- Sputter Deposition

Sputtering involves high-energy particles impacting a target or source material, resulting in the ejection of atoms from that material. These atoms are subsequently deposited onto a substrate, forming thin-film layers. In a sputtering system, a high-vacuum chamber is utilized, featuring a gas inlet for an inert gas, like argon, a pump connection, a negatively charged sputter target (cathode), and a positively charged sample (anode). Applying direct-current (DC) or alternating-current (AC or radiofrequency (RF)) excitation generates a plasma. The heightened plasma energy results in significantly greater kinetic energy of the bombarding particles compared to conventional thermal energies, leading to material removal from the sputter target.

In a study, Chang Yang et al. presented findings on the degenerate *p*-type conductivity of CuI thin films [103]. These films were grown using reactive sputtering at room temperature. In order to accomplish this, a mixture of iodine vapor and argon is utilized as the sputtering gas. It is noteworthy that the ionization of molecular iodine during sputtering substantially amplifies the chemical interaction between iodine and the sputtered copper metal, allowing for the fabrication of CuI thin films at room temperature. Additionally, the high reactivity of iodine leads to an equilibrium growth condition rich in iodine, which promotes the formation of copper vacancies and introduces holes into the CuI thin films.

Friedrich-Leonhard Schein et al. used a DC-sputtering method to deposit Cu on a glass substrate [104]. The film was exposed to iodine on a hot plate. The X-ray diffraction analysis revealed that the films consisted of polycrystalline γ-CuI and exhibited a somewhat uneven surface texture. Ultimately, they produced a transparent *p*-CuI/*n*-ZnO diode, achieving a current rectification ratio of 6 × 10^6^ at ±2 V and an ideality factor of η = 2.14.

- Magnetron sputtering

In magnetron sputtering, the bombarding source is the working gas, but the source in ion beam sputtering is an ion source. A. Voraud et al. used DC magnetron sputtering to deposit copper on glass slide substrates to produce thin film [37]. The Cu film was then dipped in I_2_ solution to produce a CuI film. The CuI thin film showed a transmittance of around 80–95% at room temperature and had a power factor of around 17 μW m^−1^ K^−2^ as its thermoelectric property. The same method was applied for deposition of CuI in making violet light-emitting diodes (LEDs) [105,106].

**Figure 14:**
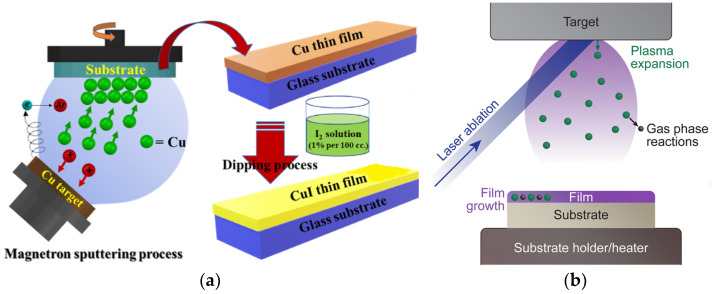
(**a**) Process of CuI thin film synthesis by magnetron sputtering of Cu followed by I_2_ reaction [37]; (**b**) an overview of the key steps in pulsed laser deposition [108].

In another report, through the combined implementation of RF magnetron sputtering and the iodine/ethanol solution method, B. Mahdy et al. have achieved the successful deposition of CuI on an inverted PVK solar cell [107]. Their investigation reveals that the underperformance of the device is primarily attributed to the presence of voids and gaps within the CuI layer (Figure 14a).

- Pulsed Laser Deposition (PLD)

Pulsed laser deposition is a physical vapor deposition technique in which a high-energy laser is focused on a target surface in a vacuum chamber and propels the expelled particles into a high-energy state, thus creating a plasma. While these particles travel from the target to the substrate, they might interact with the surrounding atmosphere, undergoing gas phase reactions. As a result, they reconstitute on the substrate’s surface, forming a thin film (Figure 14b) [108,109].

M. Rusop et al. deposited 100 nm CuI on the dye-coated TiO_2_ films on a dye-sensitized solid state solar cell at room temperature. The formation of ultrafine CuI grains was confirmed by a blue-shifted absorption spectrum of the thin film. The fill factor (FF) and power conversion efficiency were rather low (Table 2) which may be due to the deposition method of CuI, which caused the short-circuit of the charge carriers of the cell [110]. P. Storm et al. demonstrated that the film properties are highly affected by the growth temperature. Higher substrate temperatures led to notable enhancements in crystallinity compared to deposition at room temperature. When exposed to high temperatures, the surfaces displayed irregularly shaped grains, with roughness being reduced to as low as 1 nm. Additionally, the samples demonstrated an excellent transmittance of up to 90% in the visible spectrum [111]. At room temperature, 80% transmittance was reported [112].

## 3. Conclusions

In summary, this work reviews the research on the deposition methods of CuI thin film as an advanced materials for solar cells and transistors and LEDs in recent years. The coating methods are categorized into wet (solution-processed) and neat (dry) methods. Solution-processed methods, such as methods containing solvents, are classified into a variety of methods, such as spin coating, spray coating, drop casting, chemical bath deposition, and different printing techniques.

Although spin coating is an easy and widely used technique to produce thin films, unlike spray coating, it does not provide a uniform layer over large areas. To our knowledge, the best PCE (17.6%) achieved so far is for PSC when a 60 nm CuI HTL was spray-coated in an FTO/c-TiO_2_/MAPbI_3_/CuI/Au device. Drop casting, as a self-spreading phenomenon, is well aligned for industrial deposition. In SILAR and CBD techniques, the same aqueous precursors are used, and CuI is produced as the product of the reactions during different steps. Moreover, a variety of substrates, such as insulators, semiconductors, or metals, can be used in these methods.

Printing techniques that play a crucial role in the development and manufacturing of solar cells and displays include ink-jet, transfer, and spray printing for ink containing CuI dissolved in a solvent. They can produce microscale films and are currently used in the display industry. Within wet processing techniques, the doctor blade approach and spray deposition method are considered highly promising for cost-effective, large-scale, and flexible device fabrication. The advantages of the doctor blade method can be outlined as follows: (1) it is typically employed in ambient environments; (2) it boasts enhanced material efficiency; (3) it can prepare larger surfaces with varying thicknesses.

Although wet methods are simpler and cost effective, there is more thickness control in dry methods, and absence of non-toxic solvents is an advantage.

Dry methods include powder pressing and physical vapor deposition techniques, such as vacuum deposition, sputter deposition, and pulsed laser deposition methods. For deposition of CuI by PVD techniques, there are two major methods to go with: directly depositing CuI and depositing Cu on the substrate and using a solid–gas reaction. The latter γ-CuI films have a very rough surface morphology leading to a frosted glass-like appearance, rendering them far from truly transparent films. It is noteworthy to mention that applying different temperatures affects the transparency and conductivity of the films as well.

The CVD technique is compatible with various other processing methods. It is worthwhile to investigate their potential for creating innovative copper-based hole-transport materials, not limited to just CuI. Thermal evaporation methods possess the benefits of minimal pollution, ease of operation, and suitability for large-scale commercial manufacturing, in addition to continuous and reproducible films. On the other hand, scaling up this process is expensive and challenging. Therefore, utilizing non-vacuum deposition techniques could significantly decrease production expenses.

Sputtering methods show potential for upscaling, enabling the production of large-area transparent CuI thin films on inexpensive, flexible, and transparent substrates at ambient temperature. Among the various deposition methods, sputtering, pulsed laser deposition (PLD), and thermal evaporation have demonstrated lower resistivity and greater optical transmission.

Regarding thickness, it seems that a thin film with a thickness between 40 to 70 nm of CuI could produce most efficient solar cells.

In conclusion, it is believed that CuI HTM is a very promising material to replace organic HTMs and will enable low-cost, high-stability, and large-area applications of solar cells and transistors.

## Figures and Tables

**Table 2:** Photovoltaic performance of solar cells based on the copper iodide as HTL prepared by the net methods.

HTM Deposition Method	HTM Thickness [nm]	Device Configuration	V_oc_ [V]	J_sc_ [mA cm^−2^]	FF [%]	PCE [%]	Refs.
Powder pressing	1 mm	FTO-Pt-coated/CuI/PVK/m-TiO_2_/FTO	0.67	24.23	50	8.1	[94]
Thermal evaporation	70	ITO/CuI/CsSnI_3_/PCBM/BCP/Al	0.36	8.94	54	1.72	[97]
	40	FTO/CuI/PVK/PCBM/PEI-Ag	1.04	20.9	68	14.7	[113]
Thermal evaporation/solid–gas reaction	1200 (Cu)	FTO/TiO_2_/CH_3_NH_3_PbI_3_/CuI/Au	0.73	32.72	31	7.40	[100]
	120 (Cu)	FTO/TiO_2_/CH_3_NH_3_PbI_3_/CuI/Cu	0.85	22.99	47	9.24	[99]
Magnetron sputtering	440	FTO/CuI/PVK/PCBM/Au	0.49	4.6	34	0.76	[107]
Pulsed laser	100	ITO/TiO_2_/dye/CuI/Pt	0.48	12.2	48	2.8	[110]
